# Initiator-Free Recyclable Anthracene-Based Photocurable Resin Enabling Sustainable 3D Printing via Single- and Two-Photon Stereolithography

**DOI:** 10.1021/acsomega.5c09643

**Published:** 2026-02-21

**Authors:** Masaru Mukai, Wakana Miyadai, Seina Matsubara, Tomomi Aoki, Shoji Maruo

**Affiliations:** † Faculty of Engineering, 13154 Yokohama National University, 79-5 Tokiwadai, Hodogaya-ku, Yokohama 240-8501, Japan; ‡ Graduate School of Engineering Science, Yokohama National University, 79-5 Tokiwadai, Hodogaya-ku, Yokohama 240-8501, Japan

## Abstract

Single- and two-photon stereolithography are high-resolution three-dimensional (3D) printing techniques that are widely used to fabricate complex microstructures. However, the resulting structures have high cross-linking and density, which limits their recyclability. Existing approaches to addressing this issue typically rely on chain-growth polymerization, which requires photoinitiators and additional reagents for recycling. In this study, we present an anthracene-based photocurable resin that undergoes reversible photodimerization, enabling phase transitions between the solid and liquid states without photoinitiators or additives. Using this resin, we fabricated and recycled 3D microstructures via two-photon lithography, demonstrating over ten successful reprocessing cycles with minimal degradation. In addition, the resin is also compatible with single-photon microstereolithography, highlighting its versatility. These findings suggest a promising route for sustainable high-resolution additive manufacturing.

## Graphical abstract

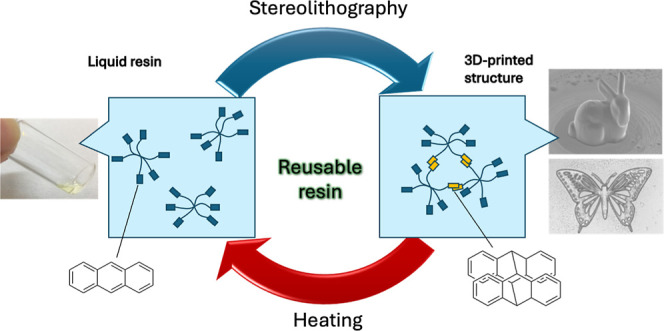

## Introduction

Three-dimensional (3D) printers are gaining attention as foundational technologies for realizing a sustainable society,^1–3^ with applications ranging from prototyping industrial products,^2,3^ custom-made medical devices for individuals,^1,2^ and to on-demand manufacturing in remote locations via 3D data sharing.^3^ Among 3D printing technologies, stereolithography offers the highest precision, and two-photon lithography based on two-photon polymerization can achieve resolutions of approximately 100 nm.^4^ In recent years, ultrahigh-resolution 3D printing using two-photon lithography has been employed not only for fabricating fine 3D structures but also for producing relatively large micropatterns and high-precision molds, such as microlens arrays, diffractive optical elements, metamaterials, cell culture scaffolds, and functional interfaces.^5,6^ However, most resins used in stereolithography are multifunctional monomers containing multiple epoxy, methacrylate, or acrylate groups, which form highly cross-linked networks that are difficult to dissolve with heat or solvents after fabrication.^7^ As a result, recycling these materials has been challenging.

To address this issue, various recyclable stereolithography resins have recently been developed for sustainable 3D printing.^8–12^ For example, Alim et al. reported a method for fabricating 3D models from thermoplastic polymers using stereolithography.^9^ However, while the resin is initially in liquid form, the fabricated product becomes solid and cannot be recycled as a liquid resin for stereolithography.

Chen et al. proposed a method to convert the material back into a usable liquid resin by breaking down the cross-linked structure via a heat exchange reaction with ethylene glycol, followed by mixing with acrylate resin to form a photocurable resin.^10^ However, this approach alters the chemical structure of the resin with each recycling cycle, resulting in darker coloration and degraded mechanical properties, indicating that complete recycling has not been achieved.

Lopez de Pariza et al. introduced an alternative recycling method based on a heat exchange reaction between thiol and isocyanate groups, enabling regeneration of the resin with its original chemical structure.^11^ This method is applicable to both single-photon microstereolithography using digital light processing (DLP) and two-photon lithography using femtosecond lasers. However, it requires the addition of fresh resin for recycling, leading to a doubling of the resin oligomer volume and increased viscosity, rendering it an incomplete recycling method. Moreover, the use of isocyanate groups raises concerns regarding resin stability and toxicity due to their high nucleophilicity.^12^

Machado et al. reported a recyclable resin based on the redox reaction of disulfides.^13^ Although they demonstrated multiple recycling cycles using single-photon microstereolithography, their system required large quantities of dithiothreitol (DTT), which may be cost-prohibitive for practical applications. Han et al. also developed recyclable resins utilizing disulfide bonds. However, their system allowed only one recycle, required resin purification prior to recycling, and exhibited changes in material properties upon recycling.^14^

Despite numerous reports on recyclable resins, only a few have achieved complete regeneration. These limitations often stem from the need to add chemical compounds during the regeneration process. Consequently, most recyclable stereolithography resins can only be recycled 1–3 times. This is because the added compounds or their reaction products must be removed from the system after regeneration to restore the resin to its original state.

By contrast, reversible reaction systems triggered by physical stimuli offer the potential for complete regeneration, provided no side reactions occur. Photodimerization is a representative reversible reaction that enables controlled chemical bonding and dissociation.^15–17^ Efforts to manipulate polymer rheological properties via photodimerization have been demonstrated.^18–20^ However, applications of such materials in stereolithography remain limited and are mostly confined to systems containing large amounts of solvents or cross-linkers.^21–23^ For instance, Gernhardt et al. used photodimerization reactions to modulate the cross-linking density of 3D-printed objects composed of radical polymerizable monomers and other cross-linking agents.^23^ However, their study did not aim to regenerate the materials. Matsuda et al. developed a coumarinated liquid biodegradable copolymer and demonstrated the fabrication of simple 2D structures via stereolithography, but did not report resin recycling.^22^

Moreover, when applied to stereolithography, solvent-containing resins tend to shrink during postprocessing (washing and drying), compromising mechanical integrity. Therefore, multifunctional monomers that remain liquid at room temperature are generally preferred for stereolithography resins.^11^ Indeed, 3D objects fabricated from hydrogels using conical molds have been shown to lose their shape, as reported by Kabb et al.^21^

In this study, we utilized the photodimerization reaction of anthracene to develop a solvent-free liquid resin that can be both photocured and thermally dissolved. We demonstrated that this material functions as a recyclable resin suitable for single- and two-photon stereolithography (Figure 1). Akiyama et al. previously reported this resin as a reusable adhesive.^24^ The material incorporates six anthracene units per molecule and remains liquid at room temperature.^24^

**Figure 1:**
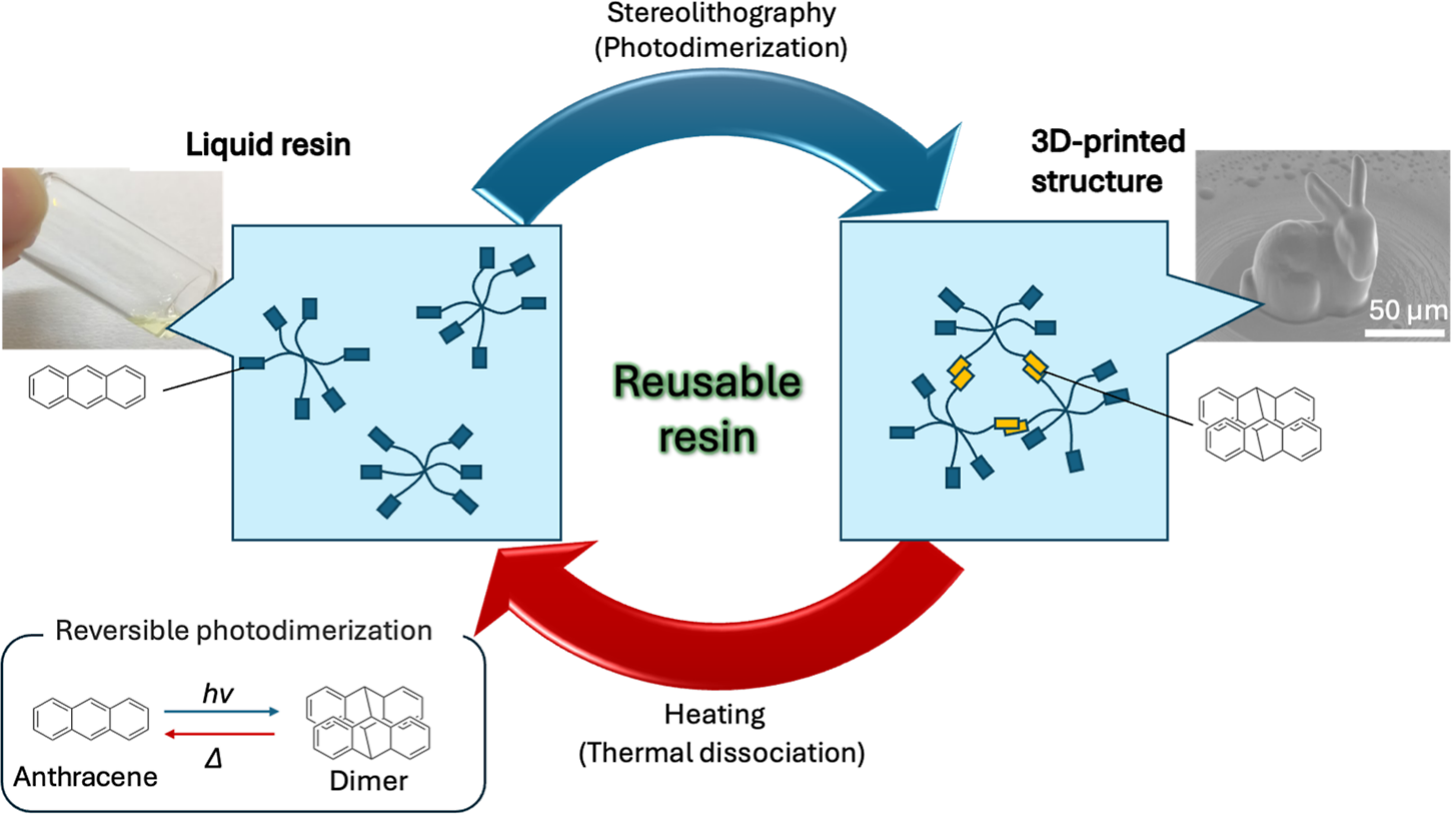
Recyclable resin using reversible photodimerization of anthracene, its use in stereolithography to create 3D objects, and regeneration of resin by heating.

Given that anthracene undergoes photodimerization to form polymer networks, this behavior aligns with the characteristics of conventional stereolithography resins—namely, multifunctional monomers that are liquid at room temperature and solidify via cross-linking reactions.^7^ Moreover, it has been shown that the material can revert to its liquid state upon heating, and the cycle of solidification and liquefaction through photoirradiation and thermal treatment can be repeated at least 5 times.^24^ This recyclability makes the material well-suited for repeated use in stereolithography.

Unlike conventional photocurable resins, which typically rely on chain polymerization mechanisms—such as radical polymerization of methacrylate resins or cationic polymerization of epoxy resins,^11^—the resin developed in this study undergoes stepwise polymerization via photodimerization. Stepwise polymerization is not commonly employed in stereolithography due to challenges in stability and kinetic control.^25^ However, anthracene-based photodimerization resins show promise for both single-photon microstereolithography and two-photon lithography.

Furthermore, while previously reported recyclable resins depend on radical polymerization and require photoinitiators,^9–12^ the resin used in this study cures via stepwise polymerization without the need for initiators. This unique feature simplifies resin formulation, eliminates contamination from additives, and enables near-complete recyclability. It was demonstrated that the resin could be recycled more than 10 times using two-photon lithography.

## Materials and Methods

### Materials

Chloroform, dichloromethane, hexane, and *N*,*N*-dimethylformamide (DMF) were purchased from Wako Pure Chemical Industries, Ltd. (Osaka, Japan). *N*,*N*-Dimethylaminopyridine, *p*-toluenesulfonic acid, *N*,*N*′-diisopropylcarbodiimide, 11-bromoundecanoic acid, d-sorbitol, and anthracene-9-carboxylic acid were obtained from Tokyo Chemical Industry Co. Ltd. (Tokyo, Japan).

### Synthesis of Recyclable Resin

The recyclable resin was synthesized following a previously reported method,^24^ where it was originally described as a reversible adhesive. Briefly, 11-bromoundecanoic acid (2.00 g), d-sorbitol (0.18 g), and *N*,*N*-dimethylaminopyridinium *p*-toluenesulfonate (2.44 g) were dissolved in 20 mL of dry dichloromethane under an argon atmosphere. The pyridinium salt was synthesized from *N*,*N*-dimethylaminopyridine and *p*-toluenesulfonic acid according to literature procedures. Next, *N*,*N*′-diisopropylcarbodiimide (1.00 g) was added, and the reaction mixture was stirred at room temperature for 19 h. The crude product was concentrated and purified by column chromatography using a dichloromethane/hexane (3:2) eluent. The solvent was removed from the purified product to yield the recyclable resin precursor. Subsequently, 0.65 g of the precursor, 1.00 g of anthracene-9-carboxylic acid, and 0.62 g of potassium carbonate were dissolved in 10 mL of dry DMF under an argon atmosphere and stirred at 80 °C for 19 h. The final recyclable resin was purified by column chromatography using chloroform as the eluent. The structure of the compound was confirmed by ^1^H NMR spectroscopy.

^1^H NMR (CDCl_3_, δ): 1.21–1.36 (m, 60H, CH_2_C*H*_2_CH_2_), 1.43 (quin, *J* = 7.6 Hz, 12H, C*H*_2_C_2_H_4_OCO), 1.50–1.62 (m, 12H, C*H*_2_CH_2_COO), 1.81 (quin, *J* = 7.0 Hz, 12H, C*H*_2_CH_2_OCO), 2.22–2.37 (m, 12H, C*H*_2_COO), 4.02 (dd, *J* = 12.1, 6.0 Hz, 1H, *H-1a*), 4.06 (dd, *J* = 12.2, 5.6 Hz, 1H, *H-6a*), 4.25 (dd, *J* = 12.2, 3.3 Hz, 1H, *H-1b*), 4.36 (dd, *J* = 11.9, 3.6 Hz, 1H, *H-6b*), 4.53–4.59 (m, 12H, C*H*_2_OCO), 5.02–5.08 (m, 1H, *H-2*), 5.19–5.25 (m, 1H, *H-5*), 7.42–7.53 (m, 24H, Ar*H*), 5.38–5.46 (m, 2H, *H-3*, *H-4*), 7.42–7.53 (m, 24H, Ar*H*), 7.98 (d, *J* = 8.3 Hz, 12H, Ar*H*), 8.01 (d, *J* = 8.2 Hz, 12H, Ar*H*), 8.48 (s, 6H, Ar*H*).

### Single-Photon Microstereolithography System

Figure 2 illustrates the schematic of the custom-built single-photon microstereolithography system used in this study.^26^ A blue semiconductor laser (Cobolt 06-MLD, Cobolt AB, Solna, Sweden) emitting at a wavelength of 405 nm served as the light source. The laser beam passed through a variable neutral density (ND) filter, allowing precise adjustment of the laser power (LP). An automatic shutter controlled the passage of the beam, enabling it to be blocked or transmitted as needed.

**Figure 2:**
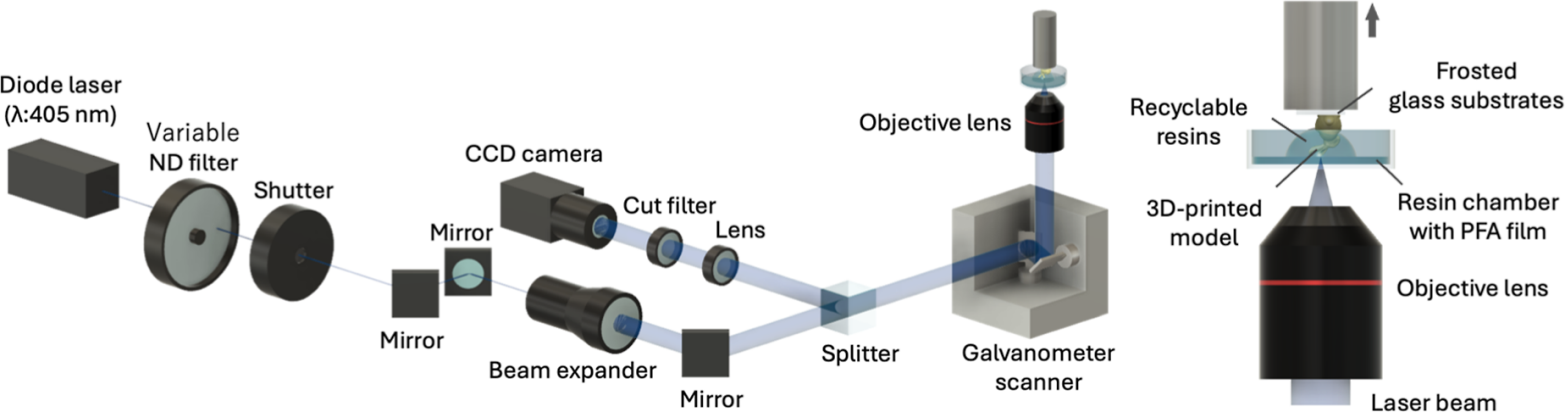
Schematic diagram of single-photon microstereolithography system.

After passing through the shutter, the beam was expanded using a beam expander and directed through a beam splitter cube and a galvanometer mirror (GM-1015, Canon Inc., Japan). The expanded beam was then focused by an objective lens (PLN4X, Olympus Corp., Tokyo, Japan) onto the interface between the recyclable resin and the upper glass substrate. The galvanometer mirror scanned the beam in the *XY* plane to define the cross-sectional geometry.

The fabrication process involved curing one layer of the cross-section, followed by incremental elevation of the stage to build up the three-dimensional structure. The reflected laser beam within the resin chamber was monitored using a charge-coupled device (CCD) camera.

To enhance adhesion between the substrate and the printed object, frosted glass substrates were used as the upper glass layer. The resin was contained within a chamber constructed from two rings fabricated using a 3D printer (Guider 2; Flashforge 3D Technology Co., Ltd., Zhejiang, China). A perfluoroalkoxy (PFA) film was sandwiched between the rings to prevent the printed resin model from adhering to the bottom of the chamber.

### Two-Photon Lithography System

Figure 3 presents a schematic of the custom-built two-photon lithography system used in this study.^27,28^ A femtosecond laser source (Mai Tai, Spectra-Physics, U.S.A.) emitting at a wavelength of 780 nm served as the excitation source. The laser beam was controlled by an automatic mechanical shutter, which allowed it to be either blocked or transmitted.

**Figure 3:**
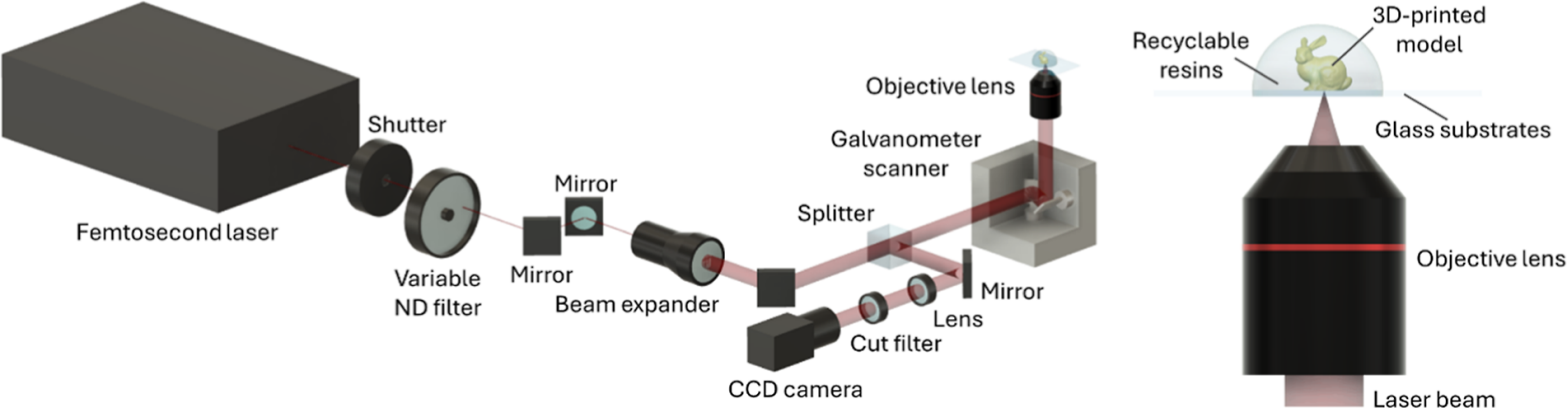
Schematic diagram of two-photon lithography system.

After passing through the shutter, the beam was directed through a variable ND filter to adjust the laser power (LP). The beam was then expanded using a beam expander and passed through a beam splitter cube. A galvanometer scanner (GM-1015, Canon Inc., Tokyo, Japan) redirected the beam upward along the *Z*-axis, and an objective lens (UPLXAPO40×, EVIDENT Corp., Japan) with a numerical aperture of 0.95 focused it onto a glass substrate mounted on a 3-axis stage (OSMS20-85­(XYZ), Sigma Koki Co., Tokyo, Japan).

The galvanometer scanner enabled *XY*-plane scanning of the laser beam, while vertical movement along the *Z*-axis was achieved using a piezoelectric stage, allowing the fabrication of three-dimensional structures. The substrate surface was monitored using a charge-coupled device (CCD) camera under transmitted illumination.

### Regeneration of Resin by Heating

The recyclable resin was first cured under light irradiation and subsequently regenerated by heating in an electric furnace. A copper plate was placed at the center of the furnace (FO810, Yamato Scientific Co., Tokyo, Japan), and a glass substrate bearing the 3D-printed object was positioned on top of the copper plate and heated. The heating time was adjusted according to the heating device and the object being formed.

### Characterization

^1^H NMR spectra were recorded using a JEOL 400 MHz spectrometer (EXC 400, JEOL Ltd., Japan), with tetramethylsilane (TMS) as the internal standard. Morphological observations were performed using a scanning electron microscope (SEM; VE-8800, Keyence Corp., Osaka, Japan), a sputter coater (SC-701, Sanyu Electron Co., Ltd., Tokyo, Japan), and an optical microscope (VHX6000, Keyence Corp., Osaka, Japan). Gold (Au) was used as the sputtering source. Mechanical strength measurements of the printed objects were conducted using a nanoindenter (TI Premier, Bruker Japan K.K., Tokyo, Japan) at two distinct positions on each sample. The tests were performed with a maximum load of 100 μN and a loading rate of 30 μN/s.

## Results and Discussion

### Characterization of Recyclable Resin

The successful synthesis of the recyclable resin was confirmed by comparing its ^1^H NMR spectra with previously reported data.^24^ The resin’s response to light and heat was then evaluated.

Initially, droplets of the resin were placed on a cover glass and tilted at 45°. The droplets exhibited high fluidity, flowing and sliding off the surface (Figure 4a). One of the reasons for selecting the compound reported by Akiyama et al.^24^ was its low viscosity, which facilitates layer formation during single-photon microstereolithography and simplifies postprocessing of printed objects. Although other studies have explored rheological control via photodimerization,^20^ those compounds typically exhibited higher viscosities and slower flow compared to the resin used here.

**Figure 4:**
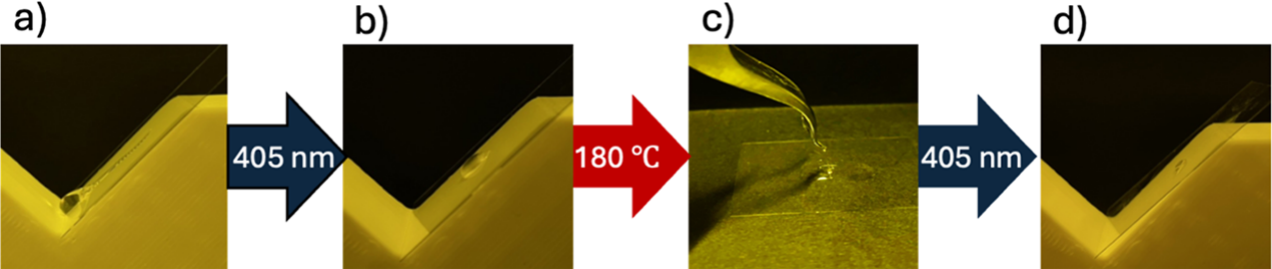
Demonstration of reversible control of physical properties of recyclable resins by light and heat. (a) Raw recyclable resin, (b) after light irradiation, (c) after heat treatment of (b), (d) after irradiating (c) with light again.

Next, the resin droplets were irradiated with a 405 nm laser beam (average intensity: 90 mW) for 30 min and then tilted at 45°. The irradiation conditions were adapted from prior literature.^24^ Postirradiation, the droplets ceased to flow, indicating a loss of fluidity (Figure 4b). This behavior is attributed to the formation of a polymer network via anthracene photodimerization. From the UV–vis spectrum, spectral changes characteristic of anthracene photodimerization upon blue laser irradiation were observed (Figure S1).

The cured material was then heated at 180 °C for 20 min, following the conditions reported by Akiyama et al.^24^ Upon cooling to room temperature, the resin regained fluidity, although it was more viscous than in its initial state (Figure 4c). This change is likely due to thermal dissociation of the anthracene dimers, which disrupts the polymer network. The incomplete recovery of viscosity suggests that thermal dissociation may not have been fully achieved. This increase in viscosity has also been reported by Akiyama et al., but despite their investigation, the reason remains unclear.^24^ They suggest the possibility of thermal decomposition, oxidation, or ester exchange reactions occurring below the detection limit. We also evaluated the NMR of heated samples but observed no clear spectral changes (Figure S2). This indicates that the side reaction is below the detection limit of NMR and that anthracene thermal dissociation is occurring efficiently.

Upon reirradiation with the 405 nm laser, the fluidity was again lost, and the material resolidified (Figure 4d). These results demonstrate that the resin’s transition between fluid and cured states can be reversibly controlled using light and heat, consistent with previous reports.^24^

### Two-Photon Lithography with the Recyclable Resin

Various stereolithography systems have been developed, including two-photon lithography using galvanometer scanners^27,28^ and 3D piezo stages,^29^ as well as single-photon microstereolithography systems employing either a single material^26,30^ or multiple materials.^31^ In this study, we employed a laser-scanning two-photon lithography system to fabricate structures using the recyclable resin (Figure 3).^28^ The laser wavelength used for 3D printing was 780 nm, which is approximately twice the maximum absorption wavelength of the resin (386 nm, Figure S1). To initially assess the feasibility of 2.5D structuring, a butterfly shaped model was printed. As shown in Figure 5, the butterfly structure was successfully fabricated by inducing polymer network formation in the laser-scanned regions under the following exposure conditions: laser power of 30 mW and scanning speed of 500 μm/s.

**Figure 5:**
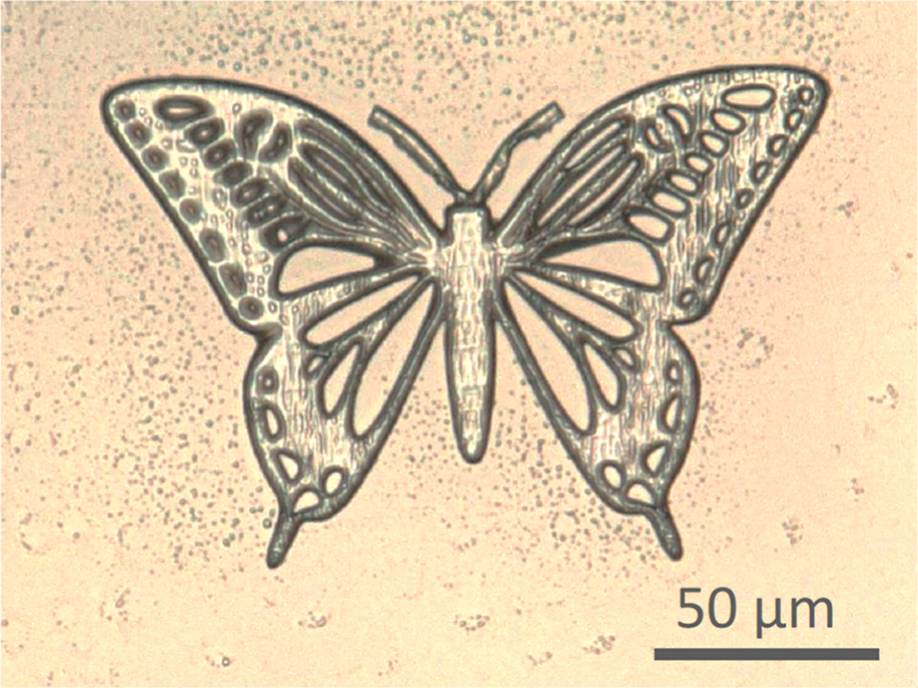
Top view of an optical microscopic image of the fabricated butterfly model by two-photon lithography.

These results demonstrate that the recyclable resin can be precisely patterned into arbitrary shapes using laser scanning, confirming its suitability for two-photon lithography.

Next, we investigated the printing accuracy based on the fabrication conditions. A single-line model supported at both ends by anchors was used to evaluate the curing line width and depth, and experiments were conducted by varying the laser power (LP) and laser scanning speed (SS) (Figures 6a,b and S3). Curing line width and depth were observed at LPs of 50 mW and 60 mW, and the lines were thicker at an SS of 50 μm/s than at 100 μm/s. The curing line width and depth tended to increase under higher LP conditions, even at the same scan speed. This trend is consistent with that of general photocurable resins based on radical polymerization, where line width and depth increase with exposure.^5,26^

**Figure 6:**
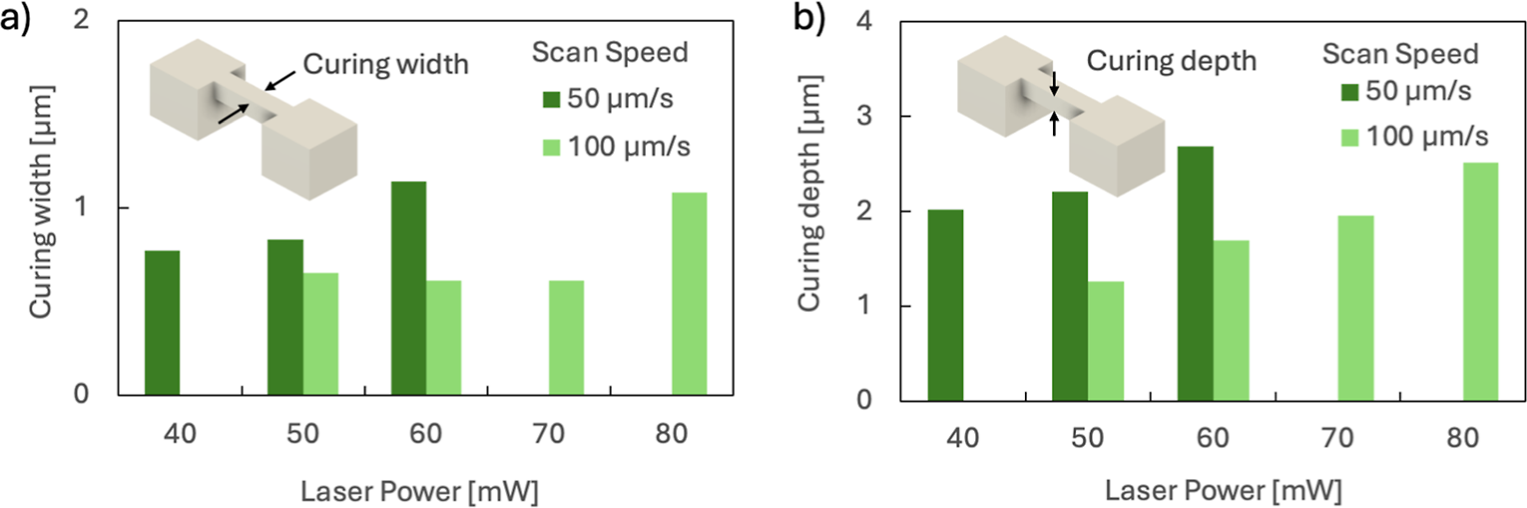
Evaluation of curing line width and depth using recyclable resin in two-photon lithography. (a) Curing line width (b) curing depth.

At an SS of 125 μm/s and LP below 30 mW, and at an SS of 250 μm/s and LP below 40 mW, no curing line was observed. This suggests the existence of a threshold, or gel point, for curing—similar to that seen in photocurable resins based on chain polymerization.^32^ At an SS of 50 μm/s and LP over 70 mW, and at an SS of 100 μm/s and LP over 90 mW, no curing line was obtained due to an explosion caused by overexposure.

Chain-growth reactions consist of multiple steps, such as initiation, propagation, and termination. In contrast, step-growth polymerization proceeds via a mechanism similar to the initial state throughout the reaction. Because recyclable resins are thought to undergo step-growth polymerization, it was expected that the exposure dose and curing line width/depth would differ from those of general chain-growth-type photocurable resins.^25^ However, the curing line width and depth observed in this study showed a similar trend to that of chain-polymerization-type resins.

In general, it is difficult to achieve localized reactions in step-growth polymerization. However, in the photodimerization reaction used in this study, polymerization can be locally activated by light. Additionally, because this is a localized polymerization reaction, efficient polymerization and dimensional control can be achieved by reducing the physical size of the reaction space. Furthermore, since the recyclable resin molecule contains six anthracene units per molecule, it is expected to lower the percolation threshold. Due to these characteristics, the minimum curing line width and curing depth achieved in these experiments were 0.61 and 1.26 μm, respectively.

Assuming the threshold for photochemical reactions is comparable to that of single-photon excitation, the square of the light intensity distribution becomes effective in two-photon absorption. Therefore, if the light intensity distribution is Gaussian, the effective excitation area shrinks to approximately 1/√2. Considering this as 1/√2 times the diffraction limit of a single photon, the minimum *z*-axis and *xy*-plane voxel sizes for two-photon systems can be expressed as follows equations (eqs 1 and 2)­

1 $$r=0.61\lambda /\left(NA\sqrt{2}\right)$$

2 $$z=2\lambda n/\left({NA}^{2}\sqrt{2}\right)$$

Here, λ represents wavelength, *n* represents refractive index, and NA represents numerical aperture. At this time, estimating the voxel size based on optical conditions (λ = 780 nm, NA = 0.95) and the resin’s refractive index (assuming *n* = 1.5) yielded *r* = 0.35 μm and *z* = 1.8 μm. In practice, the curing threshold of the resin varies depending on the exposure conditions, so the voxel size obtained experimentally differs from the theoretical value. In this experiment as well, when the exposure dose was reduced by lowering the laser intensity and increasing the scanning speed, the minimum voxel size became smaller than the theoretical value, which is consistent with the general characteristics of two-photon polymerization.

Using a laboratory-made two-photon lithography system, 3D microstructures such as microneedles and bunny models were fabricated using the recyclable resin (Figure 7). The fabrication conditions for the microneedle array and the bunny model were LP 30 mW, SS 2000 μm/s, and LP 50 mW, SS 4000 μm/s, respectively. After 3D printing, the models were rinsed with ethyl acetate to remove uncured resin. Figure 7 shows the fabrication of more complex three-dimensional structures, such as microneedles and rabbit models. In these cases, the laser scan path involves multiple overlapping exposures, making the cumulative effect of the scan history non-negligible. Therefore, the exposure conditions used for the 3D printing in Figure 7 were intentionally set lower than those used in the single-line experiments (Figure 6) to avoid excessive exposure and ensure structural fidelity. The results confirmed that 3D microstructures were successfully fabricated using the recyclable resin.

**Figure 7:**
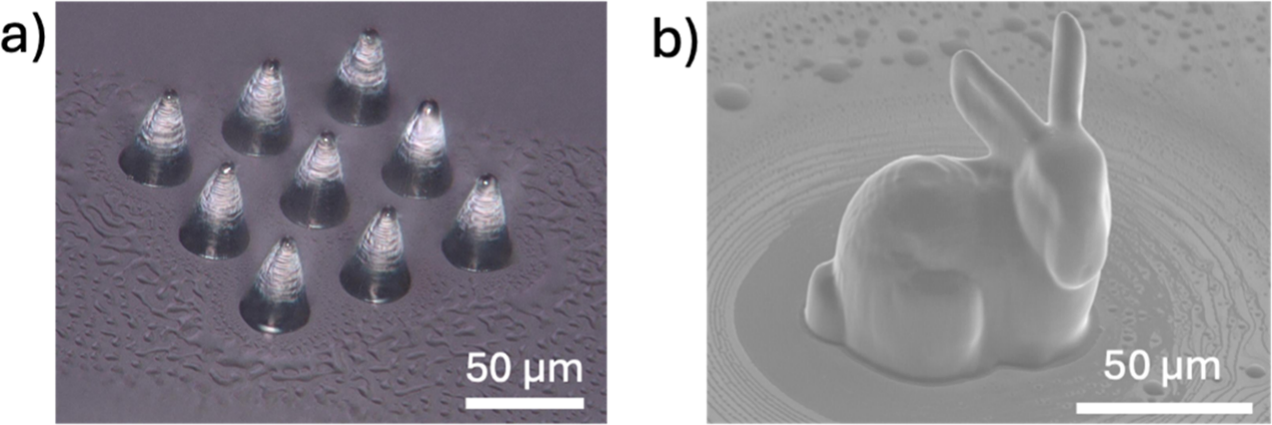
Optical microscopic image of needle array (a) and SEM image of bunny model (b) made by two-photon lithography.

### Demonstration of Light and Heat Recycling of 3D-Printed Models

To demonstrate the reusability of the recyclable resin, we conducted an experiment in which 3D-printed parts fabricated using a laboratory-made two-photon lithography system were melted by heating and then reprinted using the melted recyclable resin (Figure 8a). In this experiment, a cubic model was first fabricated using the recyclable resin (Figure 8b). After collecting the uncured resin and cleaning the resin surrounding the cubic model, the model was melted by heating at 150 °C for 15 min (Figure 8c). The melted resin was then used to fabricate a disk model. After removing the uncured resin, a 3D-printed disk was obtained (Figure 8d). These results demonstrate that the recyclable resin can be recycled by melting 3D structures fabricated via two-photon lithography.

**Figure 8:**
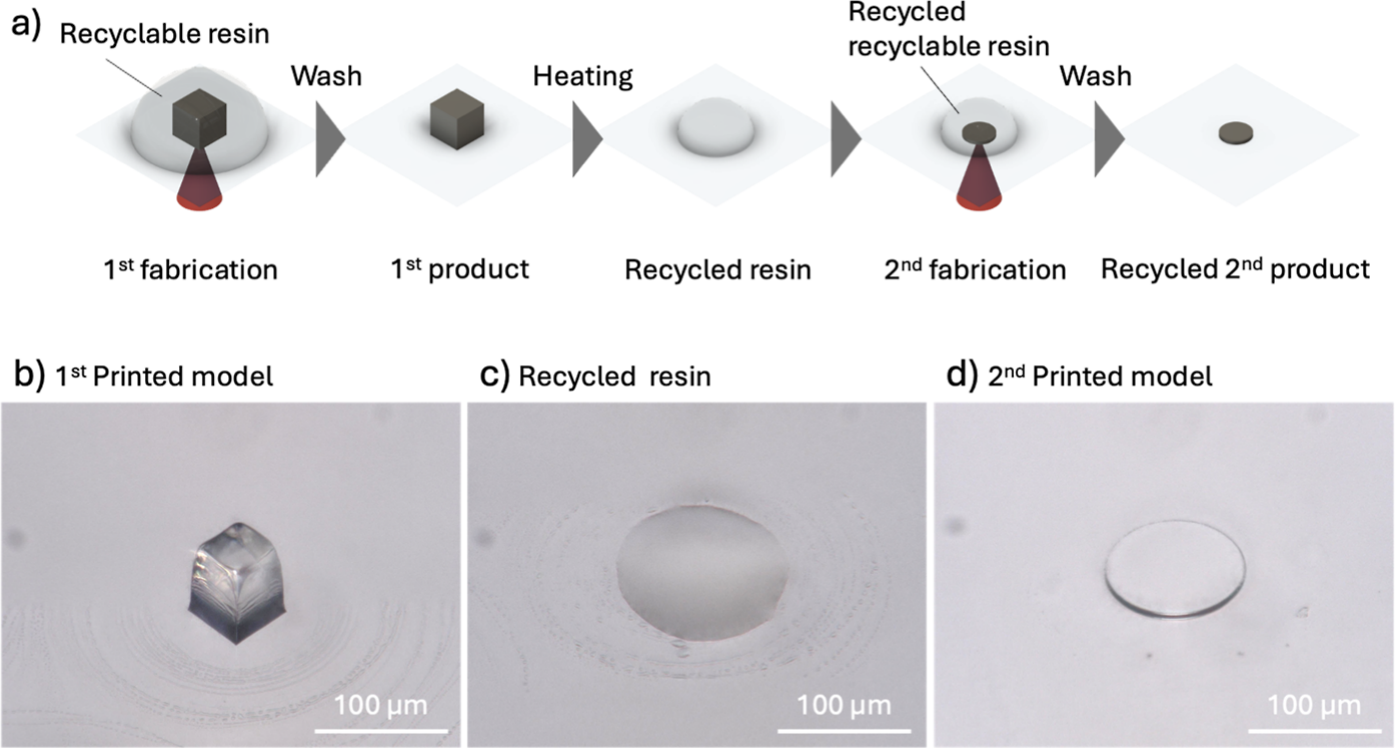
Recycling process of the recyclable resin in two-photon lithography. (a) Experimental procedure (b) 1st printed model (cube model) LP 30 mW; SS 7500 μm/s. (c) Recycled resin after thermal treatment (d) recycled 2nd printed model (disk model). LP 30 mW; SS 4000 μm/s.

In addition, the elastic modulus was evaluated using a nanoindenter with a Berkovich tip (Hysitron TI Premier, Bruker Japan K.K., Tokyo, Japan), yielding reduced elastic modulus (*E*_r_) values of 2.43, 2.66, and 2.85 GPa before recycle, after one recycle, and after two recycles, respectively. Hardness values of 110, 148, and 158 MPa before recycle, after one recycle, and after two recycles, respectively. Although slightly elevated with recycle, the cured 3D models exhibited approximately the same *E*_r_. This evaluation method, in which a cube is reprinted as a disk, requires the uncured resin to be washed away after reprinting. Consequently, the amount of resin decreases with each reproduction, limiting the ability to evaluate repeated recycling.

To demonstrate the possibility of recycling after 3D printing in droplets, the uncured resin, including 3D-printed models, was heated using an infrared heater (FPH-60/f30/12v-110w/P2m, Heat-Tech Co. Ltd., Hyogo, Japan) without washing, and the cured 3D model was melted and printed again. Heating was performed using infrared heaters for 5–5.5 min. In this method, the cured resin dissolves into the uncured resin as it is heated. This approach resembles actual recycling, where new materials are sometimes mixed with recycled ones to reduce costs and stabilize physical properties.

Specifically, we demonstrated a process in which one of the three letters of “YNU” was written in the recyclable resin, then erased by heating with an infrared heater, followed by writing the next letter repeatedly (Figure 9). Furthermore, the Video S1 shows 11 drawings made using two-photon lithography and 11 erasures by heating. At least ten rewrites were possible under these conditions. After 10 cycles of recycling, the cube-shaped specimen made from recyclable resin was evaluated using a nanoindenter, yielding a reduced modulus (*E*_r_) of 5.39 GPa and a hardness of 562 MPa. Based on prior observations, *E*_r_ increases by approximately 9% between the first and second printing cycles. Extrapolating this trend suggests that after 10 cycles, *E*_r_ becomes roughly 2.4 times the initial value. Given that the *E*_r_ of the resin molded in the first cycle was 2.43 GPa, the expected value after 10 cycles is 5.83 GPa, which closely matches the measured value. If degradation were caused solely by photochemical reactions, the effect would likely be localized, resulting in a lower measured modulus than the calculated value. However, the close agreement between the calculated and measured *E*_r_ strongly suggests that thermal effects—specifically, the heating of the entire resin during each recycling cycle—play a significant role in the material’s mechanical evolution.

**Figure 9:**
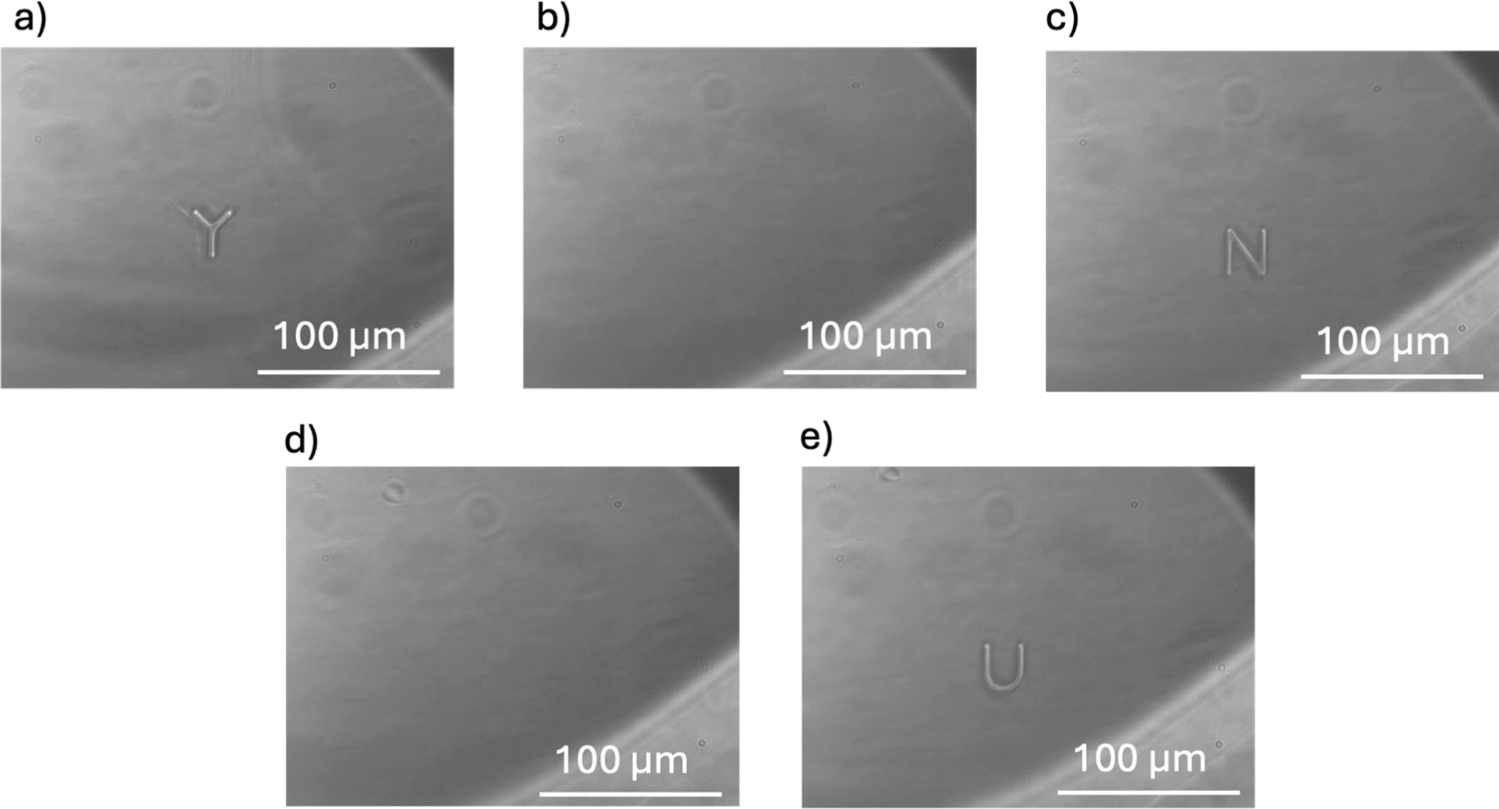
Demonstration of rewriting the letters of the alphabet using recyclable resin in two-photon lithography. (a) Drawing of “Y” (1st character), (b) erase of “Y”, (c) drawing of “N” (2nd character), (d) erase of “N”, (e) drawing of “U” (3rd character).

### Single-Photon Microstereolithography with the Recyclable Resin

Recyclable resins have also been used in single-photon microstereolithography. The 405 nm wavelength selected as the laser light source for single-photon polymerization is at the base of the peak in the spectrum of recycled resin (Figure S1), and has low absorption intensity. However, it was selected because lasers for general single-photon lithography are relatively inexpensive and this wavelength is widely used in commercially available single-photon polymerization equipment. To perform layer-by-layer printing using a laboratory-made single-photon microstereolithography system (Figure 2), we investigated the laser intensity dependence on the thickness of a single layer using a square model with sides of 500 μm (Figure 10). In the experiment, the laser scanning speed was fixed at 100 μm/s, and the laser intensity was varied to measure the thickness of the model.

**Figure 10:**
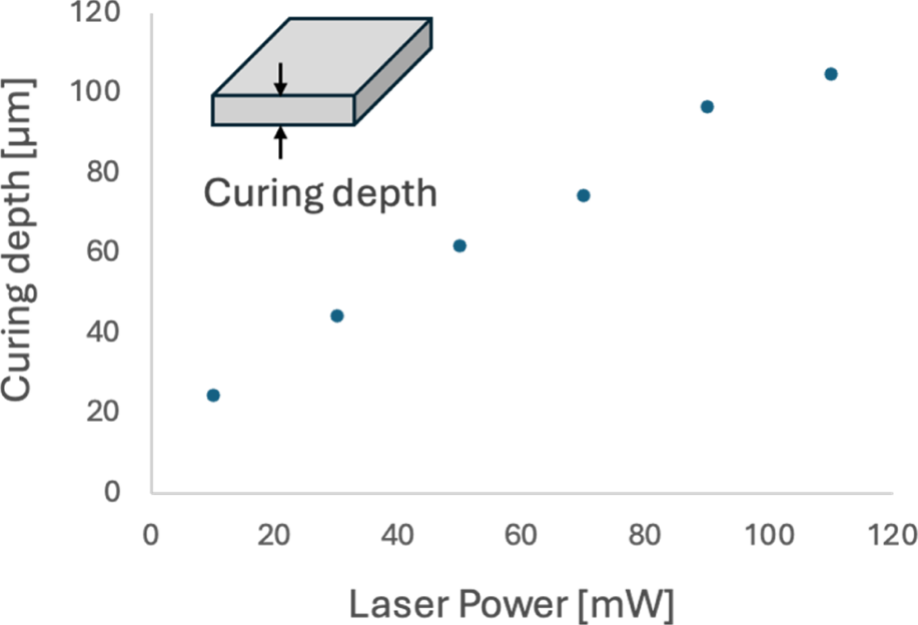
Laser power dependence of curing depth of single-layer square plates made by single-photon microstereolithography.

As a result, it was found that the thickness of the single-layer square model increased proportionally with laser intensity. The thickness could be adjusted in the range of 25 to 104 μm when the laser intensity was varied between 10 mW and 110 mW.

Next, we attempted to fabricate a 3D structure using a single-photon microstereolithography system based on a bottom-up approach. The recycled resin was originally developed as an adhesive, and its high adhesiveness may make the layer-by-layer lift-up process more difficult than with conventional photocurable resins used in stereolithography. Therefore, we tested the feasibility of the lift-up process using glass substrates, polydimethylsiloxane (PDMS), and a PFA film as the bottom substrate in the single-photon microstereolithography system shown in Figure 2.

We found that the PFA film exhibited the best performance and was suitable for the additive manufacturing of a 3D object. Using the bottom-up single-photon microstereolithography system, we employed frosted glass as the top substrate and a PFA film as the bottom of the resin chamber to print a four-tiered pyramid model (1 mm high), as shown in Figure 11. The fabrication conditions were laser power 50 mW, laser scanning speed 100 μm/s, and layer thickness 5 μm. In contrast to the relatively transparent appearance of objects fabricated using two-photon lithography, the pyramid-shaped objects exhibit a yellowish tint. This is likely because objects made via two-photon lithography are relatively small, typically less than 100 μm in height, whereas the pyramid objects measure 1000 μm overall. Their optical path length is more than 10 times greater, leading to increased light absorption or scattering, which causes them to appear colored. The results showed that the 3D model was obtained nearly exactly as designed, demonstrating that the recyclable resin can also be used in single-photon microstereolithography.

**Figure 11:**
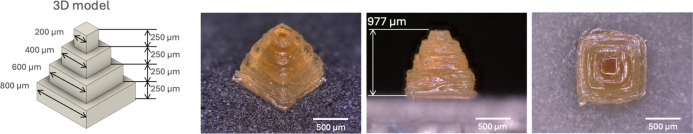
Pyramidal model made by single-photon microstereolithography.

## Conclusions

In this study, we developed a recyclable resin utilizing the reversible photodimerization of anthracene, which enables phase transitions between cross-linked solid and fluid states upon exposure to light and heat. Using two-photon lithography, we demonstrated that submicron-scale microstructures can be fabricated by reducing the exposure dose, similar to conventional chain-growth photocurable resins. The resin enabled the fabrication of complex 3D microstructures that could be thermally depolymerized and recycled for more than ten printing cycles without significant degradation.

Table 1 compares recyclable resins reported in previous literature with those developed in this study. In terms of recycling cycles, the method proposed by Han et al.^14^ is theoretically limited to a single reuse, and the number of actual reuses documented in their paper was no more than two. In contrast, our study demonstrated successful reuse over ten cycles. Moreover, other studies often require the addition of chemical additives during regeneration, involve purification steps, and are expected to incur higher costs per regeneration cycle. In contrast, our resin compound achieved over ten regeneration cycles using a simple heat treatment process, indicating a low-cost and efficient approach. Regarding printing accuracy, although direct comparisons are challenging due to differences in printing methods, our results show at least 1 order of magnitude higher accuracy than those reported in other studies. We also evaluated changes in material properties by analyzing variations in Young’s modulus during regeneration. The Young’s modulus (*E*_s_ = 1140 MPa) and Poisson’s ratio (ν_s_ = 0.07) were calculated using the Oliver–Pharr method^33^ (eq 3), assuming a diamond indenter and a typical resin Poisson’s ratio of 0.3 (ν_i_) for the printed object

3 $$\frac{1}{E_{r}}=\frac{1-{\nu_{s}}^{2}}{E_{s}}-\frac{1-{\nu_{i}}^{2}}{E_{i}}$$

**Table 1:** Comparison of Recyclable Resins for Stereolithography from Prior Literature

resin type [ref no.]	reuse cycles^a^	additives required	minimum curing width^b^	Young’s modules of 1st product	deterioration^c^
thermoplastic polymer (Alim et al.)^9^	not reusable as liquid resin	no	400 μm (DLP)^e^	75 MPa	n.d.^d^
acrylate resin with ethylene glycol (Chen et al.)^10^	multiple, but degraded (1)	yes	n.d.^d^ (DLP)^e^	2.23 GPa	–30%
thiol-isocyanate resin (Lopez de Pariza et al.)^11^	multiple (2)	yes (fresh precursor resin)	100 μm (DLP)^e^	0.77 kPa	+7%
disulfide-based resin (Machado et al.)^13^	multiple (1)	yes (large quantities of DTT)	100 μm (DLP)^e^	116 MPa	+300%
disulfide-based resin (Han et al.)^14^	only one (1)	yes (purification required)	n.d.^d^ (DLP)^e^	7 MPa	–14%
this work	multiple (over 10 time)	no	0.6 μm (TPP)^f^, 10 μm (SPP)^g^	2.22 GPa	+9%

a The numbers in parentheses indicate the number of times reuse was demonstrated in the paper.

b The text in parentheses indicates the stereolithography device’s modeling method.

c Deterioration value was calculated for ((Young’s modules of 2nd product/1st product) – 1) × 100.

d n.d.: not described.

e Digital light process (DLP).

f Two-photon polymerization (TPP).

g Single-photon polymerization (SPP).

An *E*_r_ of 2.43 GPa corresponds to a Young’s modulus of 2.22 GPa, while an *E*_r_ of 2.66 GPa corresponds to 2.43 GPa. These results indicate relatively small changes in Young’s modulus across regeneration cycles. If the modulus ratio reflects degradation, this corresponds to a 9% increase, suggesting material strengthening rather than deterioration. Although Lopez de Pariza et al.^11^ reported smaller changes in Young’s modulus during recycling, their absolute modulus values remain low compared to other studies, limiting their applicability in structural contexts. Based on these findings, the recyclable resin developed in this study demonstrates superior performance in terms of cost-efficiency, shaping accuracy, mechanical strength, and resistance to degradation.

Furthermore, we confirmed that the resin is compatible with single-photon microstereolithography, highlighting its versatility across different photopolymerization techniques. These results indicate that the resin is applicable to both two-photon and single-photon stereolithography, facilitating hybrid additive manufacturing that integrates high-resolution microfabrication with scalable 3D printing. This approach offers a promising route for the rapid fabrication of multiscale 3D architectures with dimensions ranging from submicron to millimeter. We plan to develop DLP-based printing devices capable of handling larger objects in the future.

In the future, recyclable resins may contribute to environmentally sustainable high-resolution 3D printing and the fabrication of dissolvable complex molds.

## Supplementary Material





## Notes

Figure S1: UV–vis spectra of recyclable resin; Figure S2: SEM image of curing line width and depth using recyclable; Figure S3: recycling process of the recyclable resin in single-photon lithography (PDF)
Video S1 shows the video rewriting using two-photon stereolithography and heating (MP4)

## ∥

Department of Applied Chemistry, Faculty of Science, Tokyo University of Science, 1-3 Kagurazaka, Shinjuku-ku, Tokyo 162-8601, Japan

## Abbreviations

3D: three-dimensional
DTT: dithiothreitol
DLP: digital light processing
DMF: *N*,*N*-dimethylformamide
LP: laser power
SS: laser scanning speed
CCD: charge-coupled device
TMS: tetramethylsilane
UV: ultraviolet
NMR: nuclear magnetic resonance
