# Supplementary material for: Initiator-Free Recyclable Anthracene-Based Photocurable Resin Enabling Sustainable 3D Printing via Single- and Two-Photon Stereolithography
Source: ACS Omega. 2026 Feb 21;11(9):14469–78. doi: 10.1021/acsomega.5c09643 (PMC12980437; doi:10.1021/acsomega.5c09643)
Supplement: Supplementary file 1 [file ao5c09643_si_001.pdf]

Supporting information

Initiator-free recyclable anthracene-based photocurable resin enabling sustainable 3D Printing via single- and two-photon stereolithography

Masaru Mukai [a,#,*], *Wakana Miyadai* [b], *Seina Matsubara* [b], *Tomomi Aoki* [b], *Shoji Maruo* [a,*]

[a]Faculty of Engineering, Yokohama National University, 79-5 Tokiwadai, Hodogaya-ku, Yokohama 240-8501, Japan

[b]Graduate School of Engineering Science, Yokohama National University, 79-5 Tokiwadai, Hodogaya-ku, Yokohama 240-8501, Japan

E-mail (S.M.): maruo-shoji-rk@ynu.ac.jp

E-mail (M.M.): mukai_masaru_mm@rs.tus.ac.jp

[*] Corresponding author

[#] Present address: Department of Applied Chemistry, Faculty of Science,1-3 Kagurazaka, Shinjuku-ku, Tokyo 162-8601, Japan

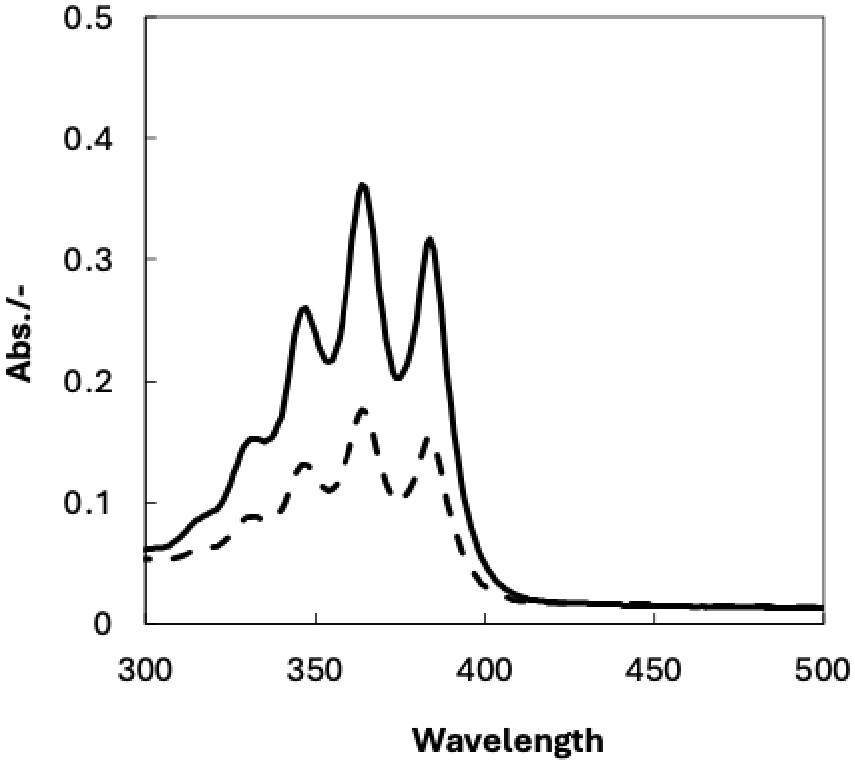

Figure S1. UV–Vis spectral changes before (solid line) and after (dashed line) 405 nm laser irradiation (119 mW) of a 11.5 µM recycled resin solution in chloroform.

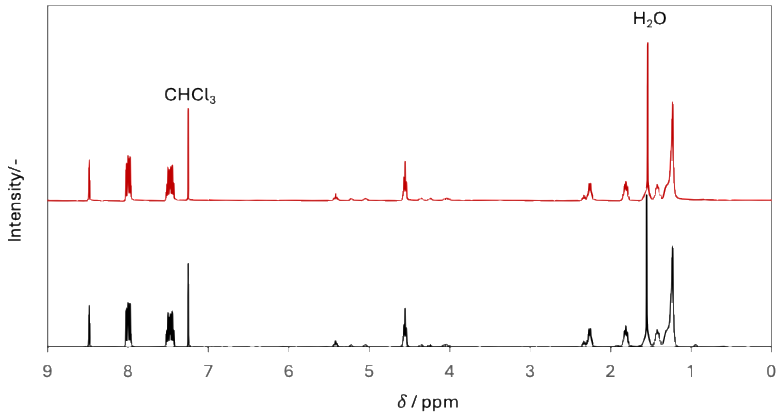

Figure S2. NMR spectra of the original recyclable resin and the resin that was heated and recycled after curing.

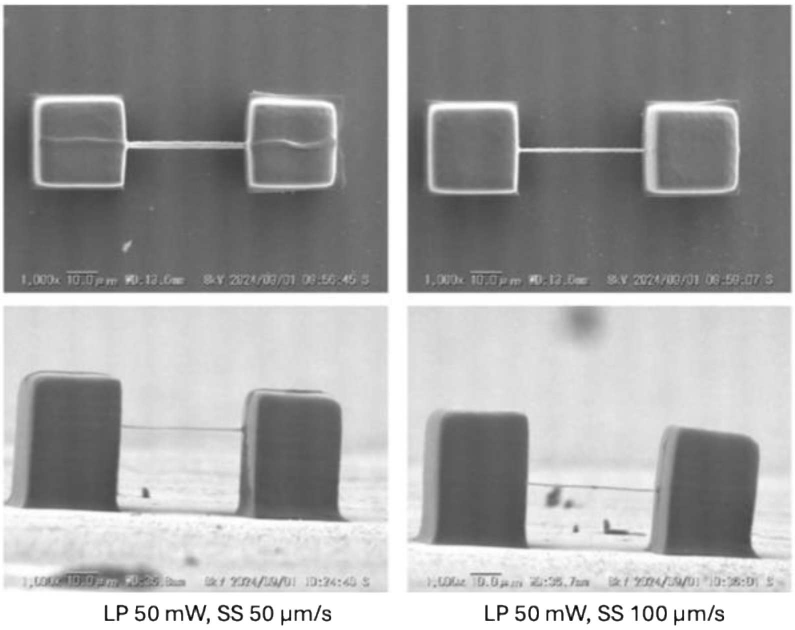

Figure S3. Example for SEM image of curing line width and depth using recyclable resin in two-photon lithography.

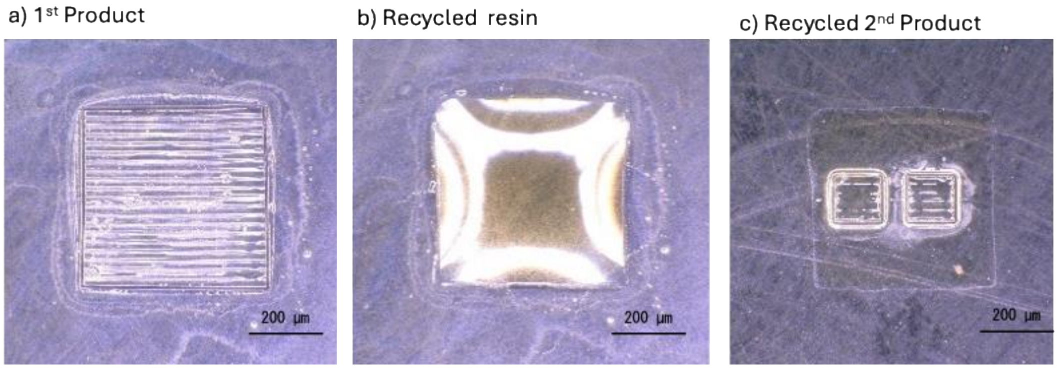

Figure S4. Recycling process of the recyclable resin in Single-photon lithography. (a) 1st Printed model (Large plate model) LP 50 mW; SS 100 µm/s. (b) Recycled resin after thermal treatment (c) Recycled 2nd Printed model (Small plate model). LP 50 mW; SS 100 µm/s.
